# Supplementary material for: Development of the PREMIUM Computerized Adaptive Testing for Measuring the Quality of Information Delivered to Patients with Severe Mental Illnesses
Source: J Clin Med. 2022 Nov 11;11(22):6687. doi: 10.3390/jcm11226687 (PMC9696867; doi:10.3390/jcm11226687)
Supplement: Supplementary file 1 [file jcm-11-06687-s001.zip › jcm-1959743-supplementary.pdf]

Supplementary Table S1. Indicators of psychometric performance.

Statistical analyses stage		Indicators
**Descriptive analysis**		- missing value rate (>70%)<br>- skewness (≤95% of response rate in one category or an absolute coefficient ≤4)<br>- inter-item correlation coefficients (≤ 0.70)<br>- Cronbach's alpha coefficient (α > 0.70)(21)
**Evaluation of IRT model assumptions**	**unidimensionality**	- 1-factor CFA, evaluated by: RMSEA ≤ 0.08 / CFI ≥ 0.95 / TLI ≥ 0.95 (22,23)<br>- EFA, with selection of the number of factors according to: Kaiser-Guttman rule (eigenvalues ≥ 1), differences in eigenvalue magnitudes (ratio > 4), scree test, parallel analysis and factor loadings (≥ 0.40) (24)<br>- bifactor structure (25), evaluated by: ECV of the general factor (≥ 60%) and omega hierarchical coefficient of the general factor (ωh ≥0.70) (26,27)
	**local independence**	residual correlations of the CFA model ≤0.25 (28,29)
	**monotonicity**	recoding of the item characteristics curves (ICC) based on the deviations of the AIC and BIC criteria between the final model (recoded items) and the initial model (no recoded items) (30,31)
**Calibration and fitting an IRT model**		- comparison with PCM, evaluated by: likelihood ratio test (32), AIC and BIC criteria (30,31)<br>- discrimination parameter ≥0.50 (33,34)<br>- goodness-of-fit evaluated by INFIT in the range [0.7-1.3] (35,36)
**Differential item functioning**		If overall DIF detected at $p<0.01$, magnitude evaluated by pseudo R^2 change (ΔR^2) according to Zumbo's classification: negligible if $\Delta R^2 < 0.13$, moderate if $0.13 < \Delta R^2 < 0.26$ and large if $\Delta R^2 > 0.26$ (37)
**CAT administration algorithm**		- accuracy: correlation coefficients (r) between CAT scores and scores for whole item banks ≥ 0.90<br>- precision: RMSE ≤ 0.30 (38)

Abbreviations: IRT item response theory / CFA confirmatory factor analysis / RMSEA root mean square error of approximation / CFI comparative fit index / TLI Tucker–Lewis index / EFA exploratory factor analysis / ECV percentage of explained common variance / PCM partial credit model / AIC Akaike information criterion / BIC Bayes information criterion / DIF differential item functioning / RMSE root mean square error / CAT computerized adaptive testing.

Supplementary Table S2. Descriptive statistics of the item bank.

Item no.	Mean ±Standard Deviation	Floor effect (%)	Ceiling effect (%)	Missing values (%)	Skewness coefficient
**I1**	2.33±1.35	14.0	22.0	2.0	-0.52
**I2**	2.05±1.41	18.0	19.0	3.0	-0.14
**I3**	2.12±1.38	16.0	18.0	6.0	-0.27
**I4**	1.87±1.58	30.0	23.0	3.0	-0.34
**I5**	1.67±1.44	30.0	14.0	0	-0.05
**I6**	2.59±1.44	15.0	34.0	2.0	-0.71
**I7**	2.94±1.32	9.0	46.0	3.0	-0.79
**I8**	1.97 ±1.42	22.0	15.0	3.0	-0.28
**I9**	2.01 ±1.47	22.0	19.0	6.0	-0.16
**I10**	2.39 ±1.47	18.0	22.0	6.0	-0.88
**I11**	2.39 ±1.41	19.0	34.0	4.0	-1.19
**I12**	2.53 ±1.52	24.0	22.0	7.0	-0.04
**I13**	1.85 ±1.55	11.0	36.0	4.0	-1.06
**I14**	2.75 ±1.34	16.0	37.0	9.0	-0.74
**I15**	2.51 ±1.56	19.0	34.0	9.0	-0.74
**I16**	1.94 ±1.52	25.0	18.0	11.0	-0.47
**I17**	2.72 ±1.37	10.0	38.0	3.0	-0.36
**I18**	2.79 ±1.36	9.0	41.0	4.0	-0.47
**I19**	3.03 ±1.08	3.0	31.0	24.0	-0.67
**I20**	1.82 ±1.58	26.0	21.0	15.0	-0.17
**I21**	1.41 ±1.24	13.0	4.0	49.0	0.07
**I22**	1.53 ±1.41	20.0	66.0	34.0	0.15

**I23**	1.77 ±1.59	29.0	23.0	5.0	0.36
**I24**	1.28 ±1.41	40.0	11.0	2.0	0.45
**I25**	1.47 ±1.51	36.0	16.0	5.0	0.07

Supplementary Table S3. Parameter estimates (discrimination and threshold) and fit statistics for the 19 items in the final item bank.

Item no.	Discrimination	Threshold 1	Threshold 2	Infit
**I1**	2.11	-0.71	0.90	1.01
**I3**	2.12	-0.50	1.07	0.93
**I5**	1.40	-0.20	1.26	0.92
**I6**	2.26	-0.82	0.67	0.84
**I7**	2.08	-0.98	0.58	0.78
**I8**	1.86	-0.48	1.00	0.84
**I9**	2.47	-0.35	0.98	0.85
**I11**	2.37	-1.23	0.43	0.88
**I12**	1.75	-0.15	0.89	0.87
**I13**	2.70	-1.15	0.47	0.83
**I14**	2.07	-0.90	0.51	0.77
**I16**	2.05	-0.72	0.87	0.89
**I18**	1.61	-0.64	0.73	0.85
**I19**	1.63	-0.93	0.71	0.87
**I20**	1.07	-0.40	1.15	0.92
**I22**	1.24	-0.07	1.37	0.97
**I23**	1.15	0.37	1.23	0.78
**I24**	1.16	0.40	1.58	0.89
**I25**	1.10	0.07	-1.20	0.91

Supplementary Table S4. DIF results.

Item no.	Sex		Age		Care setting		Main diagnosis	
	p value	ΔR²	p value	ΔR²	p value	ΔR²	p value	ΔR²
**I1**	0.556	-	0.157	-	0.627	**-**	0.074	-
**I3**	**0.004**	**0.011**	0.963	-	0.533	**-**	0.960	-
**I5**	0.058	-	0.144	-	0.079	**-**	**0.001**	**0.019**
**I6**	0.122	-	0.990	-	0.260	-	0.634	-
**I7**	0.277	-	**<0.001**	**0.019**	**<0.001**	**0.027**	0.090	-
**I8**	0.647	-	0.955	-	**0.014**	**-**	0.285	-
**I9**	0.828	-	0.876	-	**0.692**	**-**	0.535	**-**
**I11**	0.073	-	0.368	-	**0.025**	**-**	0.982	-
**I12**	0.883	-	0.335	-	0.846	-	**<0.001**	**0.020**
**I13**	0.346	-	0.477	-	0.416	-	0.855	-
**I14**	0.057	**-**	0.707	-	0.192	-	0.673	-
**I16**	**0.009**	**0.011**	0.964	-	**0.007**	**0.012**	0.184	**-**
**I18**	0.828	-	0.910	-	**<0.001**	**0.032**	0.562	-
**I19**	**0.011**	**0.009**	0.238	-	**<0.001**	**0.034**	0.237	-
**I20**	0.180	-	0.181	-	0.444	-	**<0.001**	**0.033**
**I22**	**<0.001**	**0.023**	0.149	-	**0.031**	**0.009**	**0.007**	**0.020**
**I23**	0.048	-	0.529	-	0.365	**-**	0.862	**-**
**I24**	**<0.001**	**0.020**	0.464	-	0.046	**-**	0.398	-
**I25**	0.043	**-**	0.439	-	0.107	-	**0.004**	**0.016**

Notes: Bold values indicate DIF p value <0.01. ΔR²: DIF magnitude: negligible (ΔR² < 0.13), moderate (0.13 ≤ ΔR² ≥ 0.26), or large (ΔR² ≥ 0.26)
